# On Growing Old

**Published:** 1908-11-28

**Authors:** 


### The Hospital

A Journal of

The Medical Sciences and Hospital Administration.

New Series. No. 90, Vol. IV. [No. 1162, Vol. XLV.] SATURDAY, NOVEMBER 28, 1908.

### On Growing Old.

How is it that we continue alive as long as we do? *Per contra*, how is it that, once living, we do not live for ever? These questions seem so elemental that ignorance of the answer has the appearance of being gross. However, so it is. We know neither what Life is, nor what Death is, nor how nor why the one passes into the other. This is not for want of guessing, for many centuries and much hard thinking have been devoted to this business in the realm of pure speculation. But these speculations have not advanced us a whit, and even now, when the sacrosanct problem of Life has long since been impounded by biologists and submitted to the cold contemplation of science, we are forced to confess that the secret remains remarkably well hidden. Though much has been learnt of the processes attending Life, and of the conditions governing its continuance or conclusion, what in essence it is, how it begins, and what determines the gradual failure which we call senescence, on these matters we are still pretty thoroughly in the dark. "Radiobes," the putative examples of a spontaneous generation under the influence of radium emanations, have had their day, and have left us what we were, unexplained examples of the first and greatest mystery that man has encountered—Life.

Nevertheless, impregnable as appear the obstacles which hedge in and conceal from sight this ultimate mystery of existence the collateral problem of senescence has been attacked with a good courage and not without some minor successes. Those who are scientifically concerned with senescence may be divided into two schools. Of these one considers old age to be a natural phase in the cycle of existence and no more an anomaly than the cessation of growth when adult age is attained. The other school regards senescence as a pathological phenomenon depending upon the slow deterioration of tissues under the influence of poisons, micro-chemical, or metabolic. The greatest exponent of this school is Metchnikoff. For him old age is the expression of damage inflicted upon the organism by micro-chemical poisons from the large intestine: the elixir of life, therefore, is to be found in some agent capable of inhibiting the growth and activities of the malign inhabitants of the colon. In the lactic acid bacillus, before which the normal putrefactive bacilli of the colon are found to retire, we possess, according to this conception, the natural antidote to old age.

The latest contributor to the biological theory of old age is Dr. C. S. Minot,* professor of comparative anatomy in the Harvard Medical School, who views growth, old age, and death as phases in the normal life-cycle of living matter. In the course of his arguments he presents some old facts in new dresses and in rather unexpected applications. Taking the capacity for growth as a measure of youth, he observes that over 98 per cent. of the original power of growth with which a rabbit's ovum is endowed at the moment of fertilisation has been lost by the time it is born. How rapid is the diminution in this power may be gathered from the following figures derived from the weighing of rabbit embryos preserved in alcohol. Between the ninth and the fifteenth days of intra-uterine life the average daily increment in weight of a rabbit-embryo is 704 per cent., while in the succeeding five days, from the 15th to the 20th, it has already fallen to 212 per cent. The figures as regards man are less complete, of course, but instructive. Between the third and the fourth months the human embryo increases in weight some 600 per cent., between the fourth and the fifth months 200 per cent., and so progressively less and less. It is interesting to observe that the percentage increase recorded for the fourth month of fœtal life is the same as that supplied by the whole of the first year after birth. It is quite certain then that we must cease to regard the moment of birth as the period of earliest youth, for by the time this epoch has been reached all but an insignificant proportion of the total capacity for growth has been exhausted, and the new-born infant is, relatively to his former self, an already effete and worn out young person. Whimsical as this conception appears there seems to be no escape from it. We are old at birth, and if we do not, like Gilbert's precocious baby, die

*"The Problem of Age, Growth, and Death." By Charles S. Minot. Murray. London, 1908.

enfeebled old dotards at five, we have by then very little left of that prime function of youth, the capacity for growing.

Briefly stated, the view expounded by Dr. Minot is as follows: The process of cellular evolution, which begins with fertilisation and ends with death, he calls "cytomorphosis." It begins with the undifferentiated cell and advances always in one direction—namely through differentiation to death. Capacity for growth is the most prominent characteristic of the youthful tisue, differentiation that of the adult tissue; and these two characteristics are mutually antagonistic. According to the author "growth and differentiation of the protoplasm are the cause of the loss of the power of growth." Now the most prominent attribute of senescent cells is a relative increase in the proportion borne by the protoplasm to the nucleus; ergo, senescence depends upon this increase of protoplasm. On the other hand, the power of enormous growth which the act of fertilisation confers upon an ovum is associated during segmentation with a relative overgrowth of nuclear tissue. Ergo, the essential feature of this renewal of youth, is a relative overgrowth of nuclear tissue. "The cycle of life," we are told, "has two phases, an early brief one during which the young material is produced, then the later and prolonged one in which the process of differentation goes on. I believe these are the alternating phases of life, and that, as we define senescence as an increase and differentiation of the protoplasm, so we must define rejuvenation as an increase of the nuclear material. The alternation of phases is due to the alternation in the proportions of nucleus and protoplasm." If only Dr. Minot could suggest the cause of this latter alternation!

